# Phytochemicals from Agro-Industrial By-Products for Breast Cancer Prevention and Therapy: Molecular Mechanisms and Circular Bioeconomy Perspective

**DOI:** 10.3390/ph19060934

**Published:** 2026-06-13

**Authors:** Angela Maffia, Federica Alessia Marra, Santo Battaglia, Carmelo Mallamaci, Mariateresa Russo, Adele Muscolo

**Affiliations:** 1 Dipartimento di AGRARIA, ‘Università Mediterranea’ di Reggio Calabria, Feo di Vito, 89122 Reggio Calabria, Italy; angela.maffia@unirc.it (A.M.); federica.marra@unirc.it (F.A.M.); santo.battaglia@unirc.it (S.B.); carmelo.mallamaci@unirc.it (C.M.); 2 Dipartimento di Architettura e Design (dAeD), Via dell’Università, 89124 Reggio Calabria, Italy; mariateresa.russo@unirc.it

**Keywords:** breast cancer, phytochemicals, agro-industrial by-products, polyphenols, flavonoids, circular bioeconomy, food waste valorization, nutraceuticals, green extraction technologies, PI3K/Akt pathway

## Abstract

Breast cancer remains one of the most frequently diagnosed malignancies and a leading cause of cancer-related mortality among women worldwide. The growing interest in natural bioactive compounds has highlighted plant-derived phytochemicals as promising agents for cancer prevention and adjunctive therapy due to their pleiotropic biological activities and relatively low toxicity. In parallel, increasing attention has been directed toward agro-industrial by-products generated during food processing, which represent abundant and sustainable sources of valuable phytochemicals. This review provides a comprehensive overview of recent advances in the identification, extraction, and biological evaluation of phytochemicals derived from plants and agro-industrial residues, using pomegranate (*Punica granatum*) peels, onion (*Allium cepa*) skins, and citrus by-products as representative examples of phytochemical-rich agro-industrial residues. These by-products are rich in polyphenols, flavonoids, and other secondary metabolites—including punicalagins, ellagic acid, quercetin, hesperidin, and naringin—that have demonstrated significant antioxidant, anti-inflammatory, and anticancer properties. Recent in vitro and in vivo studies indicate that these compounds can modulate key molecular pathways involved in breast cancer initiation and progression, such as oxidative stress regulation, apoptosis induction, inhibition of cell proliferation, and suppression of signaling cascades including PI3K/Akt, NF-κB, and MAPK pathways. Furthermore, the valorization of agro-industrial waste offers a sustainable strategy to recover high-value bioactive compounds while reducing environmental impact. Overall, phytochemicals obtained from plant materials and food processing by-products represent promising functional agents for breast cancer prevention and therapy, although further studies are required to improve bioavailability, elucidate mechanisms of action, and validate their clinical potential.

## 1. Introduction

### 1.1. Global Burden of Breast Cancer (Incidence, Mortality, Subtypes)

Breast cancer remains the most frequently diagnosed cancer and a leading cause of cancer-related mortality among women worldwide. According to recent global estimates, approximately 2.30 million new breast cancer cases and about 764,000 deaths were recorded in 2023, reflecting its substantial contribution to global morbidity and mortality [1]. These figures are consistent with earlier GLOBOCAN data and confirm that breast cancer accounts for nearly one in four cancer diagnoses among women globally [2,3]. Despite advances in early detection and treatment, the global burden of breast cancer continues to increase. Projections indicate that the annual number of cases may rise to approximately 3.5 million by 2050, driven by population growth, aging, and lifestyle-related risk factors [4]. Importantly, disparities persist across regions: high-income countries exhibit higher incidence rates but lower mortality due to effective screening and treatment programs, whereas low- and middle-income countries experience disproportionately higher mortality rates due to limited healthcare access and late-stage diagnosis [5].

Recent epidemiological evidence also highlights shifting trends in breast cancer demographics. Incidence rates are increasing particularly among younger women (<50 years), with an annual rise of approximately 1–1.5% in several populations [3]. Moreover, modifiable risk factors—including obesity, dietary habits, alcohol consumption, and metabolic disorders—contribute significantly to the global disease burden, accounting for over 28% of disability-adjusted life years (DALYs) associated with breast cancer [1].

Breast cancer is a biologically heterogeneous disease comprising multiple molecular subtypes with distinct clinical outcomes and therapeutic responses. Based on receptor expression and gene profiling, the main subtypes include luminal A, luminal B, HER2-enriched, and triple-negative breast cancer (TNBC) [6]. Luminal tumors, which express hormone receptors, generally exhibit better prognosis and responsiveness to endocrine therapy. In contrast, HER2-positive tumors are more aggressive but can be targeted with monoclonal antibodies such as trastuzumab. TNBC, characterized by the absence of ER, PR, and HER2, remains the most challenging subtype due to its aggressive behavior, high metastatic potential, and lack of targeted therapies [7,8].

### 1.2. Limitations of Conventional Therapies (Toxicity and Resistance)

Conventional therapeutic strategies for breast cancer—including surgery, chemotherapy, radiotherapy, endocrine therapy, and targeted therapy—have significantly improved survival outcomes. However, these approaches are frequently associated with severe limitations, particularly systemic toxicity, off-target effects, and the emergence of drug resistance.

Chemotherapy remains a cornerstone for the treatment of aggressive and advanced breast cancer, particularly TNBC. However, most chemotherapeutic agents lack selectivity and affect both cancerous and normal cells, leading to significant adverse effects such as cardiotoxicity, neurotoxicity, myelosuppression, and gastrointestinal toxicity [7,9]. Long-term complications, including irreversible cardiac damage associated with anthracyclines, further limit their clinical use.

Radiotherapy, while effective in reducing local recurrence, is also associated with late adverse effects such as tissue fibrosis, cardiovascular complications, and secondary malignancies, particularly in long-term survivors [10]. Similarly, endocrine therapies such as tamoxifen and aromatase inhibitors, although effective in hormone receptor-positive breast cancer, are linked to adverse events including thromboembolism, osteoporosis, and endocrine disturbances [11]. In addition to treatment-related toxicities, acquired endocrine resistance remains a major obstacle in the management of hormone receptor-positive breast cancer. Several mechanisms have been implicated, including mutations in the estrogen receptor gene (ESR1), activation of alternative signaling pathways such as PI3K/Akt/mTOR, and crosstalk with growth factor receptors. These alterations may reduce responsiveness to tamoxifen and aromatase inhibitors, ultimately contributing to disease progression and therapeutic failure [11].

A major clinical challenge is the development of therapeutic resistance, which significantly compromises treatment efficacy and contributes to disease recurrence and subsequent distant metastasis. Resistance mechanisms are complex and multifactorial, involving genetic mutations, epigenetic alterations, tumor heterogeneity, and activation of compensatory signaling pathways [12]. For instance, dysregulation of key pathways such as PI3K/Akt/mTOR, MAPK, and NF-κB has been strongly associated with resistance to endocrine and HER2-targeted therapies [13].

In addition, emerging evidence highlights the role of cancer stem cells and the tumor microenvironment in promoting drug resistance and tumor relapse. These cells exhibit enhanced survival mechanisms, including increased drug efflux, resistance to apoptosis, and metabolic adaptability, further complicating treatment outcomes [14].

Collectively, these limitations underscore the urgent need for alternative or complementary therapeutic strategies that are more selective, less toxic, and capable of targeting multiple oncogenic pathways simultaneously. In this context, plant-derived phytochemicals have attracted considerable attention due to their multi-targeted mechanisms of action, favorable safety profiles, and potential to overcome drug resistance.

### 1.3. Rise of Phytochemicals and Circular Bioeconomy Concepts

In recent years, there has been a growing shift toward the exploration of plant-derived phytochemicals as sustainable and effective alternatives or complements to conventional cancer therapies. This trend is driven by increasing awareness of the limitations of synthetic drugs, including toxicity, resistance, and high development costs, alongside the urgent need for environmentally sustainable solutions in biomedical and food systems [15,16]. Phytochemicals—such as polyphenols, flavonoids, terpenoids, and alkaloids—are secondary metabolites produced by plants that exhibit a wide range of biological activities, including antioxidant, anti-inflammatory, and anticancer effects. Recent studies have emphasized their multi-targeted mechanisms of action, enabling them to modulate complex signaling networks involved in cancer progression, including oxidative stress, apoptosis, and key oncogenic pathways (PI3K/Akt, NF-κB, MAPK) [17]. This pleiotropic nature makes phytochemicals particularly attractive for integrative and preventive oncology approaches.

Parallel to these biomedical advances, the concept of the circular bioeconomy has gained significant attention as a sustainable framework for resource utilization. The circular bioeconomy aims to minimize waste generation and maximize the value extracted from biological resources by promoting reuse, recycling, and recovery strategies [18]. Within this context, agro-industrial by-products—such as fruit peels, seeds, pomace, and husks—are increasingly recognized as valuable sources of bioactive phytochemicals. Food processing industries generate substantial amounts of organic waste, often rich in phenolic compounds, dietary fibers, and other functional metabolites. For instance, pomegranate peels, citrus residues, and onion skins, which are typically discarded, have been shown to contain high concentrations of punicalagins, flavonoids, and other bioactive compounds with demonstrated anticancer potential [19,20,21]. The valorization of these by-products not only contributes to sustainable waste management but also provides a cost-effective and scalable source of high-value phytochemicals. Recent advances in green extraction technologies—such as ultrasound-assisted extraction, microwave-assisted extraction, and supercritical fluid extraction—have further facilitated the efficient recovery of phytochemicals from agro-waste, aligning with the principles of green chemistry and sustainability [22,23,24]. These technologies reduce solvent use, energy consumption, and environmental impact while improving extraction yields and compound stability. Importantly, the integration of phytochemicals derived from agro-industrial by-products into nutraceuticals, functional foods, and pharmaceutical formulations represents a promising strategy to bridge the gap between sustainability and human health. This interdisciplinary approach, combining oncology, food science, and environmental sustainability, is increasingly recognized as a key direction in modern biomedical research [25,26].

### 1.4. Aim and Novelty of the Review

The present review aims to provide a comprehensive and updated overview of plant-derived phytochemicals and agro-industrial by-products as functional agents for breast cancer prevention and therapy. Specifically, this work focuses on identifying and characterizing bioactive compounds derived from selected plant sources and food processing residues, with particular emphasis on pomegranate peels (*Punica granatum*), onion skins (*Allium cepa*), and citrus by-products.

Several recent reviews have discussed the role of phytochemicals in breast cancer prevention and therapy, mainly focusing on natural bioactive compounds and their molecular mechanisms. However, limited attention has been devoted to agro-industrial by-products as sustainable sources of anticancer phytochemicals and to the integration of circular bioeconomy principles, waste valorization strategies, and green extraction technologies within the context of breast cancer prevention and treatment [7,9,15,18,24].

The novelty of this review lies in its integrative perspective, which combines molecular oncology, phytochemistry, and circular bioeconomy concepts into a unified framework. Unlike conventional reviews that primarily focus on isolated phytochemicals or single mechanisms of action, this work highlights:

- The dual value of agro-industrial by-products as both sustainable resources and sources of potent anticancer compounds;
- The multi-targeted molecular mechanisms through which phytochemicals modulate breast cancer initiation and progression;
- The role of green extraction technologies in enhancing the recovery and applicability of bioactive compounds;
- The translational potential of these compounds in nutraceuticals, functional foods, and adjunct cancer therapies.

Furthermore, this review emphasizes recent advances (2023–2026) in the understanding of phytochemical bioactivity, including their interactions with key signaling pathways, tumor microenvironment modulation, and emerging roles in epigenetic regulation and drug resistance. It also critically discusses current limitations, such as poor bioavailability, lack of clinical validation, and challenges in standardization, thereby identifying key research gaps and future directions. By integrating sustainability with biomedical innovation, this review aims to contribute to the development of novel, eco-friendly, and effective strategies for breast cancer prevention and treatment, supporting the transition toward a more sustainable and health-oriented bioeconomy.

## 2. Phytochemicals in Cancer Prevention and Therapy: Definition and Classification

Phytochemicals are a broad and heterogeneous group of naturally occurring compounds synthesized by plants as part of their adaptive and defensive strategies against environmental stress, pathogens, and herbivory [27]. In recent years, growing scientific interest has focused on their potential role in cancer prevention and treatment, owing to their ability to interact with multiple biological targets involved in tumor initiation and progression. Unlike single-target synthetic drugs, phytochemicals typically exert pleiotropic effects, modulating interconnected signaling networks such as oxidative stress responses, inflammatory pathways, apoptosis, and cell cycle regulation. Emerging evidence from studies published between 2023 and 2025 indicates that these compounds may also influence epigenetic mechanisms and tumor–host interactions, including immune modulation and microbiota-related pathways [28]. Based on their chemical structure and biosynthetic origin, phytochemicals can be categorized into several major classes. Among these, polyphenols, flavonoids, terpenoids, and alkaloids represent the most extensively investigated groups in cancer-related research.

### 2.1. Polyphenol Class

Polyphenols constitute a large and structurally diverse group of plant-derived secondary metabolites that have attracted considerable attention due to their broad spectrum of biological activities [29]. Increasing evidence supports their role in the prevention and management of chronic diseases, including cancer, as well as their application as natural antioxidants and food-preserving agents [30]. Their anticancer potential has been primarily attributed to their capacity to regulate redox homeostasis and limit oxidative damage to cellular macromolecules. However, recent research has expanded this view, demonstrating that polyphenols can also interfere with key intracellular signaling cascades, including NF-κB, PI3K/Akt, and MAPK pathways, thereby influencing cell proliferation, survival, and metastasis [31]. In breast cancer, these mechanisms are particularly relevant because they are involved in the development and progression of hormone receptor-positive, HER2-positive, and triple-negative breast cancer subtypes. Furthermore, polyphenols have been shown to participate in epigenetic regulation, affecting DNA methylation patterns, histone modifications, and non-coding RNA expression. These properties suggest a role in the early stages of carcinogenesis as well as in tumor progression [32]. Representative compounds such as curcumin, resveratrol, and epigallocatechin gallate (EGCG) continue to be investigated for their translational potential. From a nutritional perspective, polyphenols represent one of the principal contributors to the antioxidant capacity of the human diet, playing a key role in maintaining redox balance and cellular homeostasis [33]. However, despite their well-documented bioactivity, their therapeutic potential is often constrained by limited bioavailability. This limitation is primarily associated with their low solubility, extensive first-pass metabolism, and rapid systemic clearance. Consequently, polyphenols are frequently subjected to swift absorption, metabolic transformation, and subsequent excretion—predominantly via urine—resulting in reduced circulating concentrations and diminished biological efficacy [34].

### 2.2. Flavonoids

Flavonoids represent a large and structurally diverse class of plant-derived polyphenolic compounds, biosynthesized through the phenylpropanoid pathway as secondary metabolites involved in plant defense, pigmentation, and signaling processes [35]. They are widely distributed in fruits, vegetables, seeds, flowers, and plant-derived beverages such as tea and wine, and have also been identified in certain fungal species [36]. Chemically, flavonoids share a common C6–C3–C6 backbone and are classified into several major subclasses [37], including flavones, flavonols, flavanones, anthocyanins, chalcones, and isoflavones [38]. Flavanones lack the C2–C3 double bond, resulting in a saturated heterocyclic ring, whereas flavan-3-ols are characterized by the absence of both the C2–C3 double bond and the C4 carbonyl group, together with the presence of a hydroxyl group at C3 [39]. Anthocyanins are responsible for the characteristic red, purple, and blue pigmentation of many fruits and vegetables [40], whereas isoflavones are particularly abundant in leguminous plants and have attracted interest because of their phytoestrogenic properties [41].

The extensive structural diversity of flavonoids—currently exceeding 9000 identified compounds—derives from multiple modifications of the core skeleton, including hydroxylation, methylation, glycosylation, acylation, and prenylation [42]. These modifications strongly influence their physicochemical properties, such as solubility, stability, and membrane permeability, as well as their bioavailability and biological activity. In plant tissues, flavonoids are commonly present as glycosides, where sugar moieties (e.g., glucose, rhamnose, or rutinose) are conjugated to hydroxyl groups, enhancing their solubility and facilitating intracellular transport, storage, and accumulation in vacuoles [43].

Among their diverse biological activities, antioxidant capacity is one of the most extensively investigated and is closely related to their structural features, particularly the number and position of hydroxyl groups, the presence of conjugated double bonds, and the overall degree of electron delocalization [44]. Flavonoids exert antioxidant effects through multiple complementary mechanisms, including direct scavenging of reactive oxygen species (ROS), electron or hydrogen atom donation, and stabilization of free radicals via resonance. In addition, they are capable of chelating transition metal ions such as Fe^2+^ and Cu^2+^, thereby preventing the formation of highly reactive hydroxyl radicals through Fenton-type reactions [44,45]. Flavonoids also modulate endogenous antioxidant defense systems by enhancing the activity of enzymes such as superoxide dismutase, catalase, and glutathione peroxidase, while inhibiting pro-oxidant enzymes including xanthine oxidase, cyclooxygenase, lipoxygenase, and phosphoinositide 3-kinase [46]. In breast cancer, flavonoids have attracted considerable attention because of their ability to modulate pathways involved in tumor initiation and progression, including oxidative stress responses, apoptosis, cell-cycle regulation, and metastatic processes. Compounds such as quercetin, kaempferol, naringenin, hesperetin, and genistein have demonstrated antiproliferative and pro-apoptotic activities in preclinical models, including hormone receptor-positive and triple-negative breast cancer. Their multitarget mechanisms of action and relatively low toxicity also support their translational potential as adjunctive agents capable of enhancing therapeutic efficacy and potentially overcoming treatment resistance.

### 2.3. Terpenoids

Terpenoids, also known as isoprenoids, represent one of the most extensive and structurally diverse classes of natural compounds, occurring widely in plants, fungi, and certain microorganisms [47]. They are biosynthesized from five-carbon isoprene units through two distinct metabolic routes, namely the mevalonate (MVA) and methylerythritol phosphate (MEP) pathways [48]. Based on the number of isoprene units, terpenoids are conventionally classified into monoterpenoids (C10), sesquiterpenoids (C15), diterpenoids (C20), triterpenoids (C30), tetraterpenoids (C40), and higher-order polyterpenoids. Their structural diversity is further enhanced by the presence of functional groups such as hydroxyl, carbonyl, and epoxy moieties, which contribute to their biological activity [49].

The remarkable structural diversity of terpenoids arises from a wide range of enzymatic modifications, including cyclization, oxidation, rearrangement, and conjugation reactions, which generate a vast number of derivatives with distinct physicochemical and biological properties [50]. In plants, terpenoids play important ecological roles as signaling molecules and defense agents, while many are major constituents of essential oils [51]. From a pharmacological perspective, terpenoids exhibit a broad spectrum of biological activities, including antioxidant, anti-inflammatory, antimicrobial, antiviral, and anticancer effects [51]. In breast cancer research, terpenoids have attracted increasing attention because of their ability to regulate multiple pathways involved in tumor growth and progression. Their mechanisms of action include the modulation of oxidative stress, apoptosis, cell-cycle regulation, and intracellular signaling pathways associated with cancer development and metastasis. Several terpenoid compounds have demonstrated antiproliferative activity in preclinical breast cancer models, including hormone receptor-positive and triple-negative subtypes, highlighting their potential translational relevance as complementary therapeutic agents. Furthermore, their capacity to target multiple molecular pathways suggests a possible role in overcoming therapeutic resistance and improving treatment outcomes [52].

### 2.4. Alkaloids

Alkaloids constitute a large and chemically heterogeneous group of naturally occurring compounds characterized by the presence of one or more nitrogen atoms, typically incorporated within heterocyclic ring systems [53]. They are predominantly found in plants but are also produced by fungi, bacteria, and some animal species. Biosynthetically, alkaloids are generally derived from amino acids such as tryptophan, tyrosine, phenylalanine, lysine, and ornithine, although considerable structural diversification occurs during their formation [54]. Based on their chemical structure and biosynthetic origin, alkaloids are commonly classified into several major groups, including indole alkaloids, isoquinoline alkaloids, tropane alkaloids, pyridine and piperidine alkaloids, and quinoline alkaloids, among others [55].

The high structural variability of alkaloids results from extensive biochemical transformations, including methylation, oxidation, reduction, and ring rearrangements, which contribute to their wide range of physicochemical characteristics and biological functions [56]. In plants, alkaloids primarily serve as protective agents, deterring herbivores and inhibiting microbial growth due to their toxicity and pronounced physiological effects. From a biomedical perspective, alkaloids are among the most pharmacologically active natural products, exhibiting diverse bioactivities such as analgesic, antimicrobial, antimalarial, anticancer, and neuroactive effects [57]. These activities are largely attributed to their capacity to interact with specific molecular targets, including enzymes, receptors, ion channels, and nucleic acids, thereby influencing key biological processes such as neurotransmission, cell proliferation, and metabolic regulations.

## 3. Agro-Industrial By-Products as Sustainable Sources of Bioactive Compounds

Agro-industrial processing generates large volumes of residual biomass that, until recently, were primarily managed as waste. These agro-industrial residues are nutrient-rich and biodegradable by-products derived from agricultural and food-processing operations, including materials such as husks, pulp, peels, and manure [58,59]. Traditionally, they have been disposed of in landfills or used for low-value applications. However, alternative management strategies such as anaerobic digestion, composting, and microbial fermentation have increasingly been employed to convert these materials into value-added products, including biofuels, animal feed, and biodegradable materials [60].

**Figure 1:**
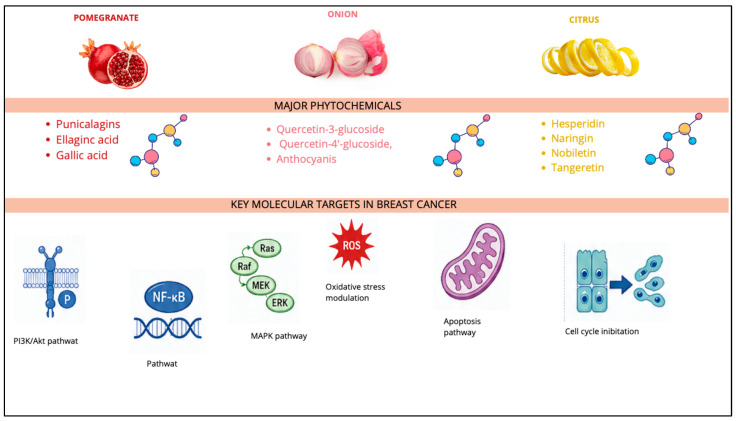
Overview of agro-industrial by-products as sustainable sources of bioactive compounds and their potential applications.

More recently, the growing demand for naturally derived bioactive compounds has further accelerated interest in these residues, promoting a shift from synthetic additives toward natural antioxidant and antimicrobial substances [61]. In this context, agro-industrial by-products are now recognized as renewable and chemically diverse matrices, often retaining a substantial fraction of the original plant’s functional constituents. Their valorization therefore represents a strategic convergence of sustainability, innovation, and industrial efficiency [62]. Figure 1 provides an overview of the principal agro-industrial by-products, their major classes of bioactive compounds, and their potential applications against breast cancer.

### 3.1. Concept of Waste Valorization

Waste valorization refers to the set of processes through which residual biomass is transformed into value-added products, rather than being discarded or used solely for low-value applications such as animal feed or energy recovery [63]. In the context of agro-industry, this concept encompasses the recovery of bioactive compounds, biopolymers, and functional ingredients that can be utilized in food, pharmaceutical, or cosmetic formulations [64]. This approach is based on the recognition that many by-products still contain high concentrations of phytochemicals due to incomplete extraction during primary processing. Valorization strategies therefore focus on selective extraction, fractionation, and stabilization of these compounds, often using innovative or environmentally friendly technologies. Importantly, waste valorization is not limited to compound recovery but also includes the development of integrated processes where multiple fractions are obtained from the same raw material, enhancing overall resource efficiency [65].

### 3.2. Types of Agro-Industrial By-Products

#### 3.2.1. Fruit Peels

Fruit peels constitute a major fraction of agro-industrial residues, particularly in juice and fresh-cut industries [66]. These outer layers are frequently richer in bioactive compounds than the edible pulp, as they play a protective role in the plant. They are notable sources of polyphenols, flavonoids, essential oils, and dietary fibers, as well as structural polysaccharides such as pectin [67].

Due to their composition, fruit peels are increasingly exploited for the extraction of antioxidant and antimicrobial compounds, as well as for the production of functional ingredients with technological properties, such as gelling and emulsifying agents. Their relatively homogeneous composition and high availability make them particularly suitable for scalable valorization processes [68].

The chemical composition of fruit peels varies depending on botanical origin, but several consistent patterns can be identified. Grape peels, commonly present in pomace generated during winemaking, are characterized by a high content of phenolic constituents, including anthocyanins responsible for pigmentation, proanthocyanidins, flavonols such as quercetin and kaempferol, and phenolic acids such as gallic and hydroxycinnamic acids [69]. They also represent an important source of stilbenes, particularly resveratrol, which has been widely investigated for its biological activity.

Citrus-derived residues, and especially bergamot peels (*Citrus bergamia*), are notable for their complex flavonoid profile. These include flavanones such as naringin and eriocitrin, as well as more structurally unique compounds such as brutieridin and melitidin, which have attracted attention due to their potential lipid-lowering effects [70]. In addition, citrus peels contain limonoids such as limonin and nomilin, as well as essential oils rich in monoterpenes, primarily limonene [71]. More generally, citrus peels from various species contain a combination of flavanone glycosides, including hesperidin and naringin, together with polymethoxylated flavones such as nobiletin and tangeretin. These compounds, along with essential oils, contribute to the broad biological activity associated with citrus by-products [72].

Pomegranate peels are distinguished by their exceptionally high concentration of hydrolyzable tannins, particularly punicalagin, which can constitute a major fraction of their dry weight. They also contain relevant amounts of ellagic acid, gallic acid, and flavonoids including rutin and hesperidin [73]. This composition contributes to their strong antioxidant capacity and makes them one of the most studied fruit by-products in the context of bioactive recovery.

Onion outer layers represent another valuable source of bioactive compounds, especially flavonols. Quercetin and its glycosylated derivatives, such as quercetin-3-glucoside and quercetin-4′-glucoside, are the predominant constituents. In pigmented varieties, anthocyanins such as cyanidin derivatives are also present, further contributing to their antioxidant potential [74].

Overall, fruit peels provide a diverse array of bioactive molecules, including polyphenols, flavonoids, tannins, and terpenoids, many of which are associated with antioxidant, antimicrobial, and anti-inflammatory effects. This chemical richness supports their valorization as functional ingredients and highlights their potential role in replacing synthetic additives with naturally derived alternatives in various industrial applications [75].

#### 3.2.2. Seeds

Seeds represent another important category of by-products generated during fruit and vegetable processing [76]. Although often discarded, they are characterized by a dense nutritional and phytochemical profile. Seeds typically contain lipophilic bioactives, including unsaturated fatty acids, tocopherols, phytosterols, and lipid-soluble antioxidants, in addition to phenolic compounds and proteins [77].

From a valorization perspective, seeds are commonly used for the extraction of high-quality oils and bioactive fractions with potential applications in nutraceuticals and pharmaceuticals. Furthermore, protein-rich seed residues can be further processed to obtain bioactive peptides, highlighting the potential for multi-step valorization pathways [78].

#### 3.2.3. Pomace

Pomace is a composite by-product generated after the mechanical extraction of juice, oil, or other primary products. It typically consists of a mixture of skins, seeds, and residual pulp, resulting in a chemically heterogeneous matrix [79]. Despite undergoing processing, pomace retains a substantial fraction of the original bioactive compounds, including phenolics, carotenoids, fibers, and minor lipids [80]. Its complex composition makes pomace particularly attractive for integrated valorization approaches, where different extraction techniques can be applied sequentially to recover multiple classes of compounds. This characteristic supports its use as a multifunctional feedstock for both bioactive recovery and material applications [81].

### 3.3. Environmental and Economic Benefits

The valorization of agro-industrial by-products provides significant environmental advantages by reducing the accumulation of organic waste and associated environmental impacts. The diversion of these materials from landfills or uncontrolled disposal mitigates greenhouse gas emissions, limits nutrient leaching, and reduces the risk of soil and water contamination [82].

From an economic standpoint, the conversion of low-value residues into high-value bioactive ingredients contributes to process intensification and diversification of revenue streams. This approach enhances the profitability and competitiveness of agro-industrial operations by maximizing the use of raw materials. In addition, the development of new products derived from by-products can stimulate innovation and open access to emerging markets, particularly in the health-related sectors [83].

### 3.4. Link to Circular Economy and Green Chemistry

The valorization of agro-industrial residues is closely aligned with the principles of the circular economy, which promotes the continuous use of resources through recycling, reuse, and regeneration. In this context, by-products are reintegrated into production systems as secondary raw materials, reducing dependence on virgin resources and minimizing waste generation [84].

At the same time, the application of green chemistry principles ensures that valorization processes are designed to minimize environmental impact. This includes the use of safer solvents, reduction in energy consumption, and implementation of efficient extraction techniques such as ultrasound-assisted, microwave-assisted, or supercritical fluid extraction [85].

The integration of circular economy and green chemistry concepts supports the development of sustainable biorefinery models, in which agro-industrial biomass is processed through cascading steps to obtain multiple valuable products. This systemic approach not only enhances resource efficiency but also contributes to a more resilient and environmentally responsible agro-industrial sector [86].

## 4. Extraction and Green Recovery Technologies

Efficient extraction technologies are essential for the recovery of bioactive phytochemicals from agro-industrial by-products. Conventional methods such as solid–liquid extraction and Soxhlet extraction remain widely used because of their robustness and reproducibility [87,88,89,90,91,92,93]. However, these techniques are often associated with long extraction times, high solvent consumption, and possible degradation of thermolabile compounds [93].

To overcome these limitations, several green extraction technologies have been developed, including ultrasound-assisted extraction (UAE), microwave-assisted extraction (MAE), and supercritical fluid extraction (SFE). These approaches improve extraction efficiency while reducing solvent use, processing time, and environmental impact [94,95,96,97,98,99,100,101,102,103,104,105,106,107].

UAE enhances mass transfer through acoustic cavitation, promoting cell wall disruption and improved release of intracellular compounds [95,96] MAE relies on microwave-induced heating, enabling rapid extraction and reduced energy consumption [97,100,101]. SFE, particularly using supercritical CO_2_, offers high selectivity and solvent-free extracts while preserving thermolabile bioactive compounds [103,104,105].

Despite their advantages, advanced extraction technologies still present important industrial challenges, including high equipment costs, scale-up limitations, and process optimization requirements. A comparative overview of the main extraction methods, operational principles, advantages, limitations, and sustainability aspects is summarized in Table 1.

## 5. Key Agro-by-Products and Their Phytochemical Profiles

Agro-industrial by-products represent a sustainable and economically viable source of bioactive phytochemicals with demonstrated relevance in cancer prevention and therapy [109]. These residues, often discarded during food processing, are particularly rich in polyphenols, flavonoids, and other secondary metabolites with antioxidant, anti-inflammatory, and antiproliferative properties. Increasing attention has been directed toward their valorization, especially in the context of breast cancer, where multiple phytochemicals have shown the ability to modulate signaling pathways involved in tumor initiation and progression [110,111]. Among these, pomegranate peels, onion skins, and citrus peels stand out due to their high phytochemical density and industrial availability.

### 5.1. Pomegranate Peels (Punica granatum)

Pomegranate peels are a major by-product of juice processing and are exceptionally rich in hydrolyzable tannins, particularly punicalagins, along with ellagic acid and its derivatives. These compounds exhibit strong antioxidant capacity and have been widely studied for their anticancer properties. Punicalagins contribute significantly to the inhibition of oxidative stress and inflammation, both of which are implicated in breast carcinogenesis [112]. Ellagic acid has been shown to modulate key molecular pathways, including apoptosis induction and cell cycle arrest in breast cancer cells [113].

The bioactivity spectrum of pomegranate peel extracts extends beyond antioxidant effects, encompassing anti-proliferative, anti-metastatic, and hormone-modulating activities. Notably, studies have demonstrated their ability to inhibit aromatase activity, a critical enzyme in estrogen-dependent breast cancer [19]. These multifaceted mechanisms position pomegranate peel phytochemicals as promising candidates for both chemoprevention and adjunct therapy.

### 5.2. Onion Skins (Allium cepa)

Onion skins, typically discarded during processing, are among the richest natural sources of quercetin and its glycosides, along with other flavonoids. Quercetin is a well-characterized bioactive compound with potent antioxidant, anti-inflammatory, and anticancer properties. It has been shown to inhibit proliferation and induce apoptosis in breast cancer cell lines through modulation of signaling pathways such as PI3K/Akt and MAPK [114].

An important advantage of onion skins lies in the relative stability of their flavonoids compared to those in edible portions, making them suitable for extraction and industrial applications. Additionally, their low moisture content and high phenolic concentration facilitate efficient recovery of bioactives using green extraction technologies [115]. These features enhance their potential as cost-effective sources of functional ingredients for nutraceutical and pharmaceutical formulations targeting breast cancer.

### 5.3. Citrus Peels (e.g., Orange, Lemon)

Citrus peels are abundant by-products of the juice industry and are rich in flavanones such as hesperidin and naringin, as well as polymethoxylated flavones. These compounds have demonstrated significant anticancer activity, including inhibition of cell proliferation, induction of apoptosis, and suppression of metastasis in breast cancer models [116]. Hesperidin and naringin, in particular, have been associated with modulation of oxidative stress and estrogen signaling pathways.

From an industrial perspective, citrus peels are widely available and scalable, making them attractive for large-scale extraction of bioactive compounds. Their widespread production ensures a consistent supply chain, supporting their integration into functional foods and therapeutic formulations. Furthermore, advances in extraction technologies have improved the recovery and bioavailability of these phytochemicals, enhancing their applicability in cancer prevention strategies [117]. Their multitarget activity is particularly relevant because simultaneous regulation of interconnected signaling pathways may help overcome compensatory mechanisms associated with tumor progression and drug resistance [118,119,120,121,122].

## 6. Molecular Mechanisms Against Breast Cancer

Phytochemicals derived from plants and agro-industrial by-products exert anticancer activity through the modulation of multiple molecular pathways involved in breast cancer initiation, progression, and metastasis. Their pleiotropic effects include regulation of oxidative stress, induction of apoptosis, inhibition of cell proliferation, suppression of metastatic processes, and modulation of major oncogenic signaling pathways such as PI3K/Akt, NF-κB, and MAPK [13]. In breast cancer models, phytochemicals have demonstrated antioxidant and pro-apoptotic activities, induction of cell cycle arrest, suppression of epithelial–mesenchymal transition (EMT), inhibition of angiogenesis, and modulation of metastatic processes [123,124,125,126,127,128,129,130,131]. A comparative overview of the principal molecular mechanisms and signaling pathways modulated by phytochemicals in breast cancer is summarized in Table 2.

## 7. Evidence from In Vitro and In Vivo Studies

A substantial body of experimental evidence supports the anticancer potential of phytochemicals derived from plants and agro-by-products in breast cancer models. Both in vitro and in vivo studies have demonstrated that these compounds can inhibit tumor growth, induce apoptosis, and interfere with metastatic processes [132]. Importantly, these studies provide mechanistic insights and help establish dose–response relationships, as well as the potential for synergistic interactions with conventional chemotherapeutic agents. The use of well-characterized breast cancer cell lines and animal models has been instrumental in validating these effects and advancing phytochemicals toward translational applications [133].

### 7.1. Studies in Breast Cancer Cell Lines

In vitro investigations using human breast cancer cell lines such as MCF-7 (estrogen receptor-positive) and MDA-MB-231 (triple-negative) have been widely employed to evaluate the anticancer activity of phytochemicals [134]. These models allow detailed assessment of cellular responses, including proliferation, apoptosis, oxidative stress, and signaling pathway modulation.

Phytochemicals such as quercetin, ellagic acid, and hesperidin have demonstrated significant antiproliferative effects in both MCF-7 and MDA-MB-231 cells. These compounds inhibit cell viability in a dose-dependent manner, induce apoptosis through caspase activation, and modulate key signaling pathways such as PI3K/Akt and MAPK [126]. In triple-negative breast cancer cells, which are typically more aggressive and resistant to therapy, flavonoids have been shown to suppress migration and invasion by targeting epithelial–mesenchymal transition (EMT) markers [135].

Additionally, in vitro studies have highlighted the role of phytochemicals in modulating oxidative stress within cancer cells. By altering intracellular ROS levels, these compounds can trigger cytotoxic effects selectively in malignant cells while preserving normal cell viability [124].

### 7.2. Evidence from Animal Models

In vivo studies using animal models provide critical validation of the anticancer effects observed in vitro. Xenograft and chemically induced breast cancer models in rodents are commonly used to assess tumor growth inhibition, metastasis, and systemic toxicity [136].

Phytochemicals have demonstrated the ability to reduce tumor volume, inhibit angiogenesis, and suppress metastatic spread in these models. For instance, flavonoid-rich extracts and isolated compounds have been shown to downregulate proliferative markers and angiogenic factors such as vascular endothelial growth factor (VEGF), while promoting apoptosis in tumor tissues [137]. Furthermore, animal studies confirm that these compounds can modulate immune responses and improve the tumor microenvironment, contributing to their overall anticancer efficacy [130].

Importantly, in vivo studies also provide insights into pharmacokinetics, bioavailability, and safety profiles, which are essential for the development of phytochemical-based therapeutics.

### 7.3. Dose–Response Relationships

Understanding dose–response relationships is crucial for evaluating the therapeutic potential of phytochemicals. Both in vitro and in vivo studies have demonstrated that the anticancer effects of these compounds are concentration-dependent. Variations in concentration may influence antioxidant activity, apoptosis induction, cell-cycle arrest, and the modulation of intracellular signaling pathways involved in cancer progression [123].

Dose optimization is particularly important due to the variability in bioavailability and metabolic stability of phytochemicals. Recent studies emphasize the need for appropriate formulation strategies, such as nanoencapsulation, to enhance their therapeutic efficacy and achieve optimal dosing in biological systems [138].

### 7.4. Synergistic Effects with Chemotherapy

One of the most promising aspects of phytochemicals is their ability to act synergistically with conventional chemotherapeutic agents. Combination treatments have been shown to enhance anticancer efficacy while reducing drug resistance and toxicity [139].

For example, flavonoids such as quercetin have been reported to sensitize breast cancer cells to chemotherapeutic drugs by modulating apoptosis-related proteins and inhibiting survival pathways such as PI3K/Akt and NF-κB [140]. This synergistic interaction can lead to enhanced cytotoxicity in cancer cells while allowing lower doses of chemotherapeutic agents, thereby minimizing adverse side effects.

Additionally, phytochemicals may help overcome multidrug resistance by inhibiting drug efflux transporters and altering cellular signaling networks involved in resistance mechanisms [141]. These findings support the potential integration of phytochemicals into combination therapy strategies for improved breast cancer management.

## 8. Bioavailability, Pharmacokinetics, and Delivery Challenges

Despite the promising anticancer potential of plant-derived phytochemicals, their clinical application is often limited by unfavorable pharmacokinetic properties, including poor aqueous solubility, limited intestinal absorption, rapid metabolism, and fast systemic elimination [142,143]. In addition, interactions with the food matrix and gut microbiota may significantly influence compound stability, release, metabolism, and bioactivity [144,145]. To improve therapeutic efficacy, several delivery strategies have been developed, including nanoformulations, encapsulation systems, and controlled-release approaches. Liposomes, polymeric nanoparticles, solid lipid nanoparticles, nanoemulsions, and biopolymer-based carriers may enhance solubility, stability, cellular uptake, and gastrointestinal resistance of phytochemicals such as quercetin, ellagic acid, curcumin, hesperidin, and naringin [146]. A summary of the principal bioavailability limitations and the main delivery strategies proposed to overcome these challenges is reported in Table 3.

Although these delivery systems have shown promising preclinical results, the development of standardized, reproducible, and clinically validated formulations remains a major challenge [98]. Further studies are needed to optimize formulation parameters, evaluate long-term safety, and determine whether enhanced bioavailability translates into meaningful anticancer effects in humans.

## 9. Clinical Evidence and Translational Potential

Although numerous in vitro and in vivo studies support the anticancer activity of phytochemicals against breast cancer, clinical evidence remains limited. Most available data derive from experimental models using breast cancer cell lines such as MCF-7, T47D, SK-BR-3, and MDA-MB-231, or from animal models designed to evaluate tumor growth, oxidative stress, apoptosis, and metastatic behavior [147]. These studies provide valuable mechanistic insights but cannot fully reproduce the complexity of human breast cancer biology.

Current clinical investigations involving phytochemicals are generally focused on dietary polyphenols, curcumin, green tea catechins, resveratrol, quercetin, and soy isoflavones. Some trials have evaluated their effects on biomarkers related to inflammation, oxidative stress, hormone metabolism, quality of life, or treatment-related toxicity. However, evidence specifically addressing phytochemicals derived from agro-industrial by-products remains scarce [148]. In particular, clinical studies on pomegranate peel, onion skin, and citrus peel extracts in breast cancer prevention or therapy are still insufficient.

A major challenge in translating preclinical findings into clinical applications is the limited pharmacokinetic performance of many phytochemicals. Numerous compounds exhibit poor aqueous solubility, low intestinal absorption, extensive first-pass metabolism, and rapid systemic elimination, resulting in low bioavailability and reduced tissue exposure. Consequently, concentrations that demonstrate anticancer activity in vitro are often difficult to achieve in vivo through dietary intake or conventional supplementation. These limitations have stimulated growing interest in advanced delivery systems, including nanoformulations, encapsulation technologies, and phytochemical combinations designed to improve stability, absorption, and therapeutic efficacy [138,143,149].

The translational potential of these compounds is therefore promising but not yet fully established. A major limitation is the discrepancy between concentrations effective in experimental models and those achievable in human tissues after dietary intake or oral supplementation. Moreover, many studies use purified compounds at high doses, whereas real-world applications are more likely to involve complex extracts, functional foods, or nutraceutical formulations [149].

Safety represents another critical issue. Although phytochemicals are often perceived as safe because of their natural origin, their biological activity may vary depending on dose, formulation, duration of use, and patient characteristics. Potential interactions with chemotherapy, endocrine therapy, targeted drugs, or anticoagulants should be carefully evaluated. Regulatory aspects also need consideration, particularly when phytochemical-rich extracts are intended for use as nutraceuticals, functional foods, or adjunct therapeutic agents [139]. Another important limitation is the lack of robust clinical validation. While many phytochemicals have shown promising anticancer effects in experimental breast cancer models, evidence from well-designed randomized clinical trials remains scarce. Existing human studies are often characterized by small sample sizes, short intervention periods, heterogeneous formulations, and the use of surrogate biomarkers rather than clinically relevant endpoints. Furthermore, regulatory classification of phytochemical-based products varies considerably between countries, creating additional challenges for quality control, standardization, efficacy assessment, and clinical implementation [139].

Overall, the transition from preclinical promise to clinical application requires well-designed human studies, standardized extracts, robust biomarkers, and clear evaluation of efficacy, safety, and drug–nutrient interactions.

## 10. Integration into Functional Foods and Nutraceuticals

Phytochemicals recovered from agro-industrial by-products offer promising opportunities for the development of functional foods and nutraceuticals with potential applications in health promotion and breast cancer prevention. By-products such as pomegranate peels, onion skins, and citrus peels are particularly rich in bioactive compounds including punicalagins, ellagic acid, quercetin, hesperidin, and naringin, which may be incorporated into food matrices or nutraceutical formulations to enhance their functional value [150,151,152]. These phytochemicals can be integrated into beverages, bakery products, cereals, snacks, dietary supplements, and encapsulated powders. However, several technological challenges remain, including compound stability during processing and storage, low bioavailability, and undesirable sensory properties such as bitterness, color alteration, and odor [151,152,153]. Encapsulation and microencapsulation strategies may improve stability, gastrointestinal resistance, and controlled release of bioactive compounds [154]. Consumer acceptance and regulatory compliance also represent important aspects for successful commercialization. Increasing demand for sustainable and health-promoting ingredients is creating favorable conditions for the valorization of agro-industrial by-products within the functional food and nutraceutical sectors [155]. A comparative overview of the principal agro-industrial by-products, their phytochemical composition, potential applications, and technological challenges is summarized in Table 4.

## 11. Sustainability and Circular Bioeconomy Perspective

The recovery of phytochemicals from agro-industrial by-products represents an important application of circular bioeconomy principles within the health and nutraceutical sectors, summarized in Table 5. Valorization of fruit peels, seeds, pomace, and other food-processing residues contributes to waste reduction, improved resource efficiency, and generation of high-value bioactive compounds [86,156]. In addition to environmental benefits, agro-industrial waste valorization may promote industrial symbiosis by integrating food, pharmaceutical, nutraceutical, cosmetic, and bioenergy sectors into more sustainable value chains [156]. However, the real sustainability of these processes depends on multiple factors, including extraction efficiency, energy consumption, solvent recovery, transportation, and waste management, highlighting the importance of life-cycle assessment (LCA) approaches [157,158].

## 12. Challenges, Knowledge Gaps, and Future Directions

Despite the growing interest in plant-derived phytochemicals and agro-industrial by-products for breast cancer prevention and therapy, several translational challenges remain unresolved. Most available evidence is still based on in vitro and preclinical models, which cannot fully reproduce tumor heterogeneity, immune interactions, and metabolic complexity observed in humans. Moreover, many in vitro studies employ phytochemical concentrations that are substantially higher than those achievable through dietary intake or conventional supplementation in humans. As a result, the promising anticancer effects observed under experimental conditions may not necessarily translate into comparable clinical outcomes. In addition, in vitro models do not adequately account for factors such as absorption, metabolism, bioavailability, tissue distribution, and interactions with the tumor microenvironment, all of which can significantly influence therapeutic efficacy.

In addition, differences in extraction methods, phytochemical composition, and experimental conditions often generate inconsistent and poorly reproducible results.

Another major limitation is the lack of standardization. Phytochemical profiles may vary according to plant species, cultivar, geographical origin, agricultural practices, storage conditions, and processing methods, making comparison among studies difficult. Furthermore, clinical validation remains limited, highlighting the need for well-designed randomized controlled trials using standardized extracts, clinically relevant doses, and validated biomarkers, particularly across different breast cancer subtypes.

The multitarget activity of phytochemicals represents both an opportunity and a challenge. While simultaneous modulation of oxidative stress, inflammation, apoptosis, metabolism, and signaling pathways may improve therapeutic efficacy, it also complicates mechanistic interpretation and regulatory approval. Future studies may benefit from integrated omics approaches, including genomics, transcriptomics, proteomics, metabolomics, and microbiomics, to better characterize molecular targets and interindividual responses. In parallel, artificial intelligence and machine learning may support the identification of bioactive compounds, optimization of extraction processes, and prediction of synergistic interactions. Although numerous phytochemicals have demonstrated anticancer activity in breast cancer models, direct comparisons of their relative efficacy remain limited. Polyphenols such as curcumin, resveratrol, quercetin, and epigallocatechin gallate (EGCG) are among the most extensively investigated compounds and have consistently shown antiproliferative, pro-apoptotic, and anti-metastatic effects across multiple experimental models. In contrast, evidence regarding several terpenoids and alkaloids remains comparatively limited and is often restricted to preclinical investigations. However, meaningful comparisons are complicated by substantial differences in study design, experimental conditions, phytochemical concentrations, and breast cancer subtypes. Future studies should therefore adopt standardized methodologies to enable more robust comparative assessments and facilitate the identification of the most promising candidates for clinical translation.

Future research should also consider sustainability and industrial feasibility. Although agro-industrial waste valorization is generally regarded as environmentally beneficial, the real sustainability of extraction and processing technologies should be quantitatively assessed through life-cycle assessment (LCA) studies, including evaluation of energy consumption, solvent recovery, transport, waste management, and overall environmental impact. Currently, only limited quantitative LCA data are available for many phytochemical recovery processes, particularly those involving emerging green extraction technologies and agro-industrial by-products. Future studies should therefore integrate standardized environmental indicators, such as carbon footprint, energy demand, water consumption, and solvent recovery efficiency, to enable more robust sustainability assessments and facilitate comparison among extraction strategies.

Such analyses will be essential to ensure that phytochemical recovery strategies are not only biologically effective but also economically and environmentally sustainable within a circular bioeconomy framework.

Although preclinical evidence is substantial, clinical evidence remains comparatively limited. Most human studies conducted to date have involved small patient cohorts, short intervention periods, and heterogeneous phytochemical formulations, making direct comparisons difficult. Available clinical investigations generally suggest favorable safety profiles and potential beneficial effects on biomarkers associated with oxidative stress, inflammation, and treatment-related adverse effects. However, robust evidence supporting efficacy in breast cancer prevention or treatment is still lacking, highlighting the need for large-scale randomized controlled trials using standardized preparations and clinically relevant endpoints.

Finally, personalized nutrition and precision oncology may represent promising future directions, as individual differences in genetics, gut microbiota composition, metabolism, lifestyle, and tumor biology are likely to influence responses to phytochemical-based interventions.

## 13. Conclusions

Plant-derived phytochemicals recovered from agro-industrial by-products represent a promising and rapidly evolving area of research at the intersection of oncology, food science, biotechnology, and sustainability. Beyond their recognized antioxidant properties, compounds such as quercetin, ellagic acid, punicalagins, hesperidin, and naringin have demonstrated the ability to modulate multiple molecular pathways involved in breast cancer progression, including oxidative stress responses, apoptosis, inflammation, cell-cycle regulation, and metastatic processes. These multitarget activities make phytochemicals attractive candidates for future preventive and adjunct therapeutic strategies.

However, the clinical translation of these findings remains challenging. Major obstacles include variability in phytochemical composition, lack of extract standardization, limited bioavailability, insufficient pharmacokinetic characterization, and the scarcity of robust clinical studies. Furthermore, the concentrations required to achieve biological activity in experimental models are often difficult to reproduce under physiological conditions, highlighting the need for more realistic and clinically relevant research approaches.

Future advances in this field will require the development of standardized extraction and characterization protocols, reproducible formulations, and innovative delivery systems capable of improving stability, absorption, and tissue bioavailability. Particular attention should also be directed toward the evaluation of phytochemical efficacy across different breast cancer subtypes, including hormone receptor-positive, HER2-positive, and triple-negative breast cancers, in order to support more personalized and mechanism-based intervention strategies.

From an industrial perspective, the successful valorization of agro-industrial residues will depend not only on biological efficacy but also on economic feasibility, scalability, regulatory compliance, and environmental sustainability. Future studies should therefore integrate techno-economic analyses and quantitative life-cycle assessment (LCA) approaches to evaluate energy consumption, solvent recovery, carbon footprint, and overall environmental impact. Such assessments will be essential to ensure that phytochemical recovery processes provide genuine sustainability benefits within a circular bioeconomy framework.

Ultimately, the convergence of phytochemistry, molecular oncology, green extraction technologies, precision nutrition, and precision oncology offers a unique opportunity to transform agro-industrial waste into high-value health-promoting resources. Achieving this goal will require multidisciplinary collaboration and stronger integration between preclinical research, clinical investigation, industrial innovation, and sustainability assessment. Such efforts may contribute to the development of next-generation phytochemical-based strategies that are not only environmentally sustainable but also clinically relevant for breast cancer prevention and management.

## Figures and Tables

**Table 1:** Comparison of conventional and green extraction technologies used for phytochemical recovery from agro-industrial by-products.

Extraction Method	Principle	Main Operational Parameters	Advantages	Limitations	Green/Sustainability Aspects	Representative Applications	Industrial Scalability
Conventional Solvent Extraction/Solid–Liquid Extraction	Diffusion and partitioning of phytochemicals between plant matrix and solvent phase [87,88]	Solvent polarity, solvent-to-solid ratio, extraction time, temperature, agitation, particle size [89]	Simple, robust, reproducible, easy to implement; suitable for a broad range of compounds [87]	Long extraction times, high solvent consumption, co-extraction of impurities, possible degradation of thermolabile compounds [90]	Limited sustainability due to intensive solvent and energy use	Recovery of polyphenols, flavonoids, tannins, and antioxidant compounds from agro-industrial residues	High
Soxhlet Extraction	Continuous solvent reflux and recycling through repeated percolation cycles [91]	Solvent type, reflux temperature, extraction duration, particle size [87]	Exhaustive extraction, high reproducibility, minimal manual intervention [92]	High energy demand, prolonged extraction time, large solvent volumes, degradation of heat-sensitive phytochemicals [93]	Low environmental sustainability because of solvent and energy requirements	Reference method for comparative phytochemical studies and validation protocols	Low
Ultrasound-Assisted Extraction (UAE)	Acoustic cavitation induces cell disruption and enhanced mass transfer [94]	Ultrasound power/frequency, extraction time, temperature, solvent type, solid-to-liquid ratio [94]	Faster extraction, reduced solvent use, improved extraction efficiency, low energy consumption [96]	Possible degradation from localized hotspots; industrial scale-up challenges [97]	Green extraction method compatible with eco-friendly solvents and reduced waste generation	Recovery of phenolics, flavonoids, and antioxidant compounds from fruit peels and agro-wastes	Moderate–High
Microwave-Assisted Extraction (MAE)	Microwave heating through dipole rotation and ionic conduction causing rapid internal heating [98]	Microwave power, extraction time, temperature, solvent type, moisture content, particle size [99]	Rapid extraction, high yield, lower solvent consumption, improved reproducibility [101]	Hotspot formation, degradation of sensitive compounds, scale-up difficulties [101,102]	Reduced solvent and energy consumption; aligned with green chemistry principles	Efficient extraction of polyphenols and bioactives from plant-derived matrices	Moderate
Supercritical Fluid Extraction (SFE)	Extraction of lipophilic compounds such as carotenoids and sterols, as well as selected flavonoids when polar co-solvents (e.g., ethanol) are used; production of pharmaceutical-grade phytochemicals [103]	Pressure, temperature, CO_2_ flow rate, co-solvent concentration, particle size [104]	High selectivity, solvent-free extracts, short extraction times, preservation of thermolabile compounds [105]	High equipment cost, operational complexity, scale-up limitations, need for co-solvents for polar compounds [107,108]	Highly sustainable technology with low solvent residues and recyclable CO_2_	Extraction of lipophilic compounds, flavonoids, carotenoids, sterols, and pharmaceutical-grade phytochemicals	High

**Table 2:** Main molecular mechanisms of phytochemicals against breast cancer.

Mechanism	Main Molecular Targets/Pathways	Biological Outcome	Representative Phytochemicals	References
Oxidative stress modulation	ROS scavenging, antioxidant enzymes	Reduced oxidative damage and genomic instability	Polyphenols, flavonoids	[123,124]
Apoptosis induction	Bax/Bcl-2 balance, caspases, cytochrome c	Cancer cell death	Quercetin, ellagic acid	[121,122]
Cell cycle arrest	Cyclins, CDKs, p21, p27	Inhibition of proliferation	Flavonoids, polyphenols	[122,123]
Anti-metastatic activity	MMP-2, MMP-9, EMT markers, VEGF	Reduced invasion, migration, and angiogenesis	Hesperidin, naringin	[124,125,126]
PI3K/Akt pathway inhibition	Akt phosphorylation	Apoptosis induction and reduced survival	Quercetin, ellagic acid	[123,127]
NF-κB pathway suppression	IκB/NF-κB signaling	Reduced inflammation and anti-apoptotic signaling	Polyphenols	[128]
MAPK pathway modulation	ERK, JNK, p38 kinases	Reduced proliferation and enhanced apoptosis	Flavonoids	[129]
Crosstalk modulation	PI3K/Akt, NF-κB, MAPK interactions	Prevention of compensatory resistance mechanisms	Multiple phytochemicals	[130,131]

**Table 3:** Main bioavailability limitations of phytochemicals and proposed delivery strategies.

Limitation	Consequence	Proposed Strategy	Representative Compounds	References
Poor aqueous solubility	Reduced intestinal absorption and low bioavailability	Nanoformulations, nanoemulsions	Curcumin, quercetin	[142,146]
Rapid metabolism (phase I/II reactions)	Reduced plasma and tissue concentration	Encapsulation and controlled-release systems	Polyphenols, flavonoids	[143,144]
Fast systemic elimination	Short biological half-life	Sustained and controlled-release delivery	Ellagic acid, hesperidin	[143,146]
Food matrix interactions	Altered stability and absorption	Optimized formulation strategies	Phytochemical-rich extracts	[144]
Gut microbiota biotransformation	Variable metabolite production and activity	Microbiota-targeted delivery approaches	Polyphenols	[145]
Low gastrointestinal stability	Reduced therapeutic efficacy	Biopolymer-based carriers (chitosan, alginate, cyclodextrins)	Naringin, quercetin	[146]

**Table 4:** Agro-industrial by-products, major phytochemicals, potential applications, and technological challenges for functional food and nutraceutical development.

Agro-Industrial By-Product	Main Phytochemicals	Potential Applications	Technological Challenges	References
Pomegranate peels (*Punica granatum*)	Punicalagins, ellagic acid	Antioxidant ingredients, nutraceutical supplements, functional beverages	Stability during processing and storage	[150,151]
Onion skins (*Allium cepa*)	Quercetin, quercetin glycosides	Functional food ingredients, encapsulated formulations, dietary supplements	Bitterness, color, and odor management	[152]
Citrus peels (*Citrus* spp.)	Hesperidin, naringin, polymethoxylated flavones	Beverage fortification, nutraceutical formulations, antioxidant additives	Bioavailability and compound stability	[151,153]
Phytochemical-rich extracts	Polyphenols, flavonoids	Bakery products, cereals, snacks, dairy alternatives	Heat and pH sensitivity during processing	[150,153]
Encapsulated phytochemicals	Polyphenols and flavonoids	Controlled-release nutraceuticals and functional foods	Scale-up and formulation standardization	[153,154]

**Table 5:** Sustainability and circular bioeconomy aspects associated with agro-industrial by-product valorization.

Sustainability Aspect	Environmental/Industrial Benefit	Potential Applications	References
Waste valorization	Reduction in landfill disposal and organic waste accumulation	Recovery of phytochemicals from food-processing residues	[86,155]
Resource efficiency	Improved utilization of agricultural biomass	Functional ingredients and nutraceutical production	[155]
Industrial symbiosis	Integration of food, pharmaceutical, cosmetic, and nutraceutical sectors	Sustainable regional value chains	[156]
Green extraction technologies	Reduced solvent and energy consumption	Sustainable phytochemical recovery	[157,158]
Life-cycle assessment (LCA)	Evaluation of environmental and economic sustainability	Process optimization and sustainability validation	[157]
Cascading biomass utilization	Minimization of waste generation and maximization of biomass value	Animal feed, compost, bioenergy, bioplastics, dietary fibers	[158]

## Data Availability

No new datasets were generated or analyzed during the current study. All information discussed in this review is derived from previously published studies cited in the manuscript.
